# Crystal structure, supra­molecular and Hirshfeld surface analysis of piperazine-1,4-diium bis­(2,5-di­bromo­benzoate)

**DOI:** 10.1107/S205698902600798X

**Published:** 2026-08-14

**Authors:** Veerappan Subha, Marimuthu Sathish, Thangavelu Balakrishnan

**Affiliations:** a PG and Research Department of Physics Government Arts College (Autonomous and affiliated to Bharathidasan University, Tiruchirappalli) Thanthonimalai Karur-639 005 Tamil Nadu India; b Crystal Growth Laboratory PG and Research Department of Physics Thanthai Periyar Government Arts and Science College (Autonomous and affiliated to Bharathidasan, University, Tiruchirappalli) Tiruchirappalli-620 023 Tamil Nadu India; Illinois State University, USA

**Keywords:** crystal structure, proton transfer, piperazine-1,4-diium cation, 2,5-di­bromo­benzoate, Hirshfeld surface analysis

## Abstract

The title salt, C_4_H_12_N_2_^2+^·2C_7_H_3_Br_2_O_2_^−^, crystallizes in the monoclinic space group *P*2_1_/*c* with *Z* = 4. The asymmetric unit comprises one-half of a piperazine-1,4-diium dication, located about an inversion center, and one 2,5-di­bromo­benzoate anion. In the dication, both nitro­gen atoms of the piperazine ring are protonated, while the 2,5-di­bromo­benzoic acid is deprotonated at the carboxyl group. The proton is transferred from the acid to the piperazine mol­ecule, resulting in the formation of the ionic salt. Charge neutrality is achieved by the presence of one dication for every two monobasic 2,5-di­bromo­benzoate anions. The piperazine ring adopts a chair conformation. The cations and anions are linked *via* N—H⋯O and N—H⋯Br hydrogen bonds, in which the carboxyl­ate O atoms and Br atoms act as acceptors. These inter­actions (N—H⋯O/Br), together with additional C—H⋯Br contacts, contribute to the cohesion of the crystal structure. The resulting supra­molecular architecture is analyzed in detail. Hirshfeld surface (HS) analysis, supported by two-dimensional fingerprint plots, indicates that H⋯O/O⋯H (47.5%) and H⋯Br/Br⋯H (34.0%) contacts are the major contributors for the cation, whereas H⋯Br/Br⋯H (31.6%) and H⋯O/O⋯H (21.4%) inter­actions dominate for the anion.

## Abstract 2

The crystal structure of piperazine-1,4-diium bis­(2,5-di­bromo­benzoate) is reported, with detailed analysis of its intra- and inter­molecular hydrogen-bonding inter­actions, supra­molecular architecture, and Hirshfeld surface analysis.

## 1. Chemical context

Piperazine is a six-membered heterocyclic aliphatic amine containing nitro­gen atoms at the 1 and 4 positions, resulting in a symmetrical structure. These nitro­gen atoms enable efficient hydrogen-bond formation, rendering piperazine a versatile organic base with high solubility in water and many organic solvents. Owing to these properties, piperazine exhibits significant biological relevance in pharmaceutical applications (Brito *et al.*, 2019 ▸; Shaquiquzzaman *et al.*, 2015 ▸) and is also widely utilized in industrial chemistry, making it a compound of considerable research inter­est. Furthermore, its ability to form stable supra­molecular assemblies via non-covalent inter­actions has attracted attention in the fields of supra­molecular chemistry and crystal engineering (Chen & Peng, 2008 ▸, 2011 ▸; Wang *et al.*, 2012 ▸; Priyanka *et al.*, 2022 ▸).

A search of the Cambridge Structural Database (CSD, Version 5.45, update of June 2024; Groom *et al.*, 2016 ▸; Ferrence *et al.*, 2023 ▸) reveals 69 structures of protonated piperazine-1,4-diium salts with substituted benzoate anions, including chloro-, amino-, nitro-, hy­droxy-, and polycarb­oxy derivatives. The crystal structures and supra­molecular features of related piperazine-1,4-diium salts with various counter-anions have been reported previously (Allu *et al.*, 2023 ▸; Roshini, & Jayalakshmi, 2023 ▸; Roy *et al.*, 2023 ▸; Sun *et al.*, 2023 ▸; Banik *et al.*, 2016 ▸).

As part of our ongoing studies on piperazine-based systems, including the hydrated 2:1 adduct of piperazine-1,4-diium-3,5-di­nitro-2-oxidobenzoate with piperazine (Subha *et al.*, 2022*a* ▸), and 4-(2-meth­oxy­phen­yl)piperazine-1-ium 3,5-dinitro­salicyl­ate (Subha *et al.*, 2022*b* ▸), we report herein the crystal structure of piperazine-1,4-diium bis­(2,5-di­bromo­benzoate). The study includes detailed structural characterization, analysis of inter­molecular inter­actions, and HS analysis (Spackman & Jayatilaka, 2009 ▸) to explore the supra­molecular architecture.
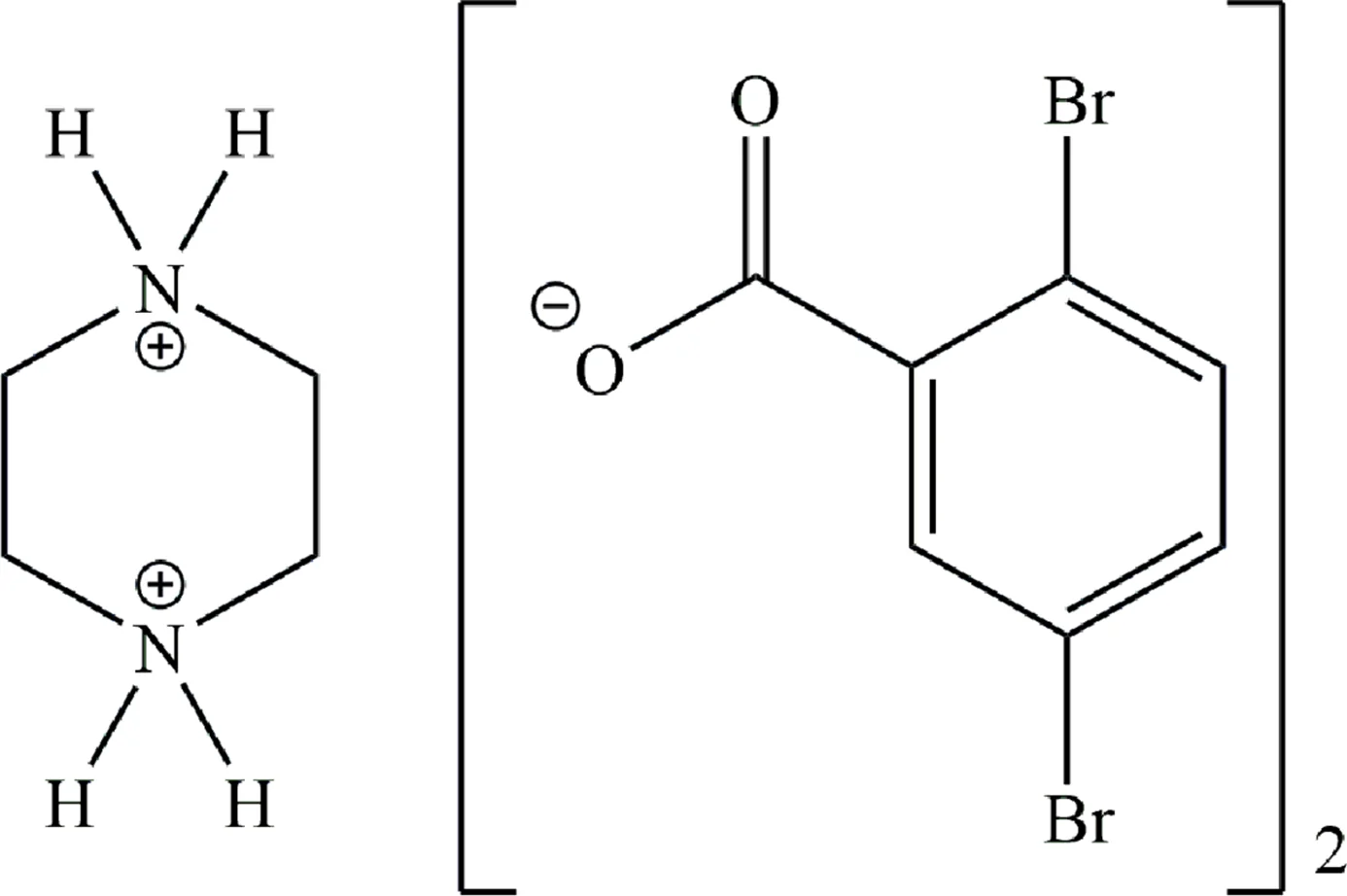


## 2. Structural commentary

**Figure 1:**
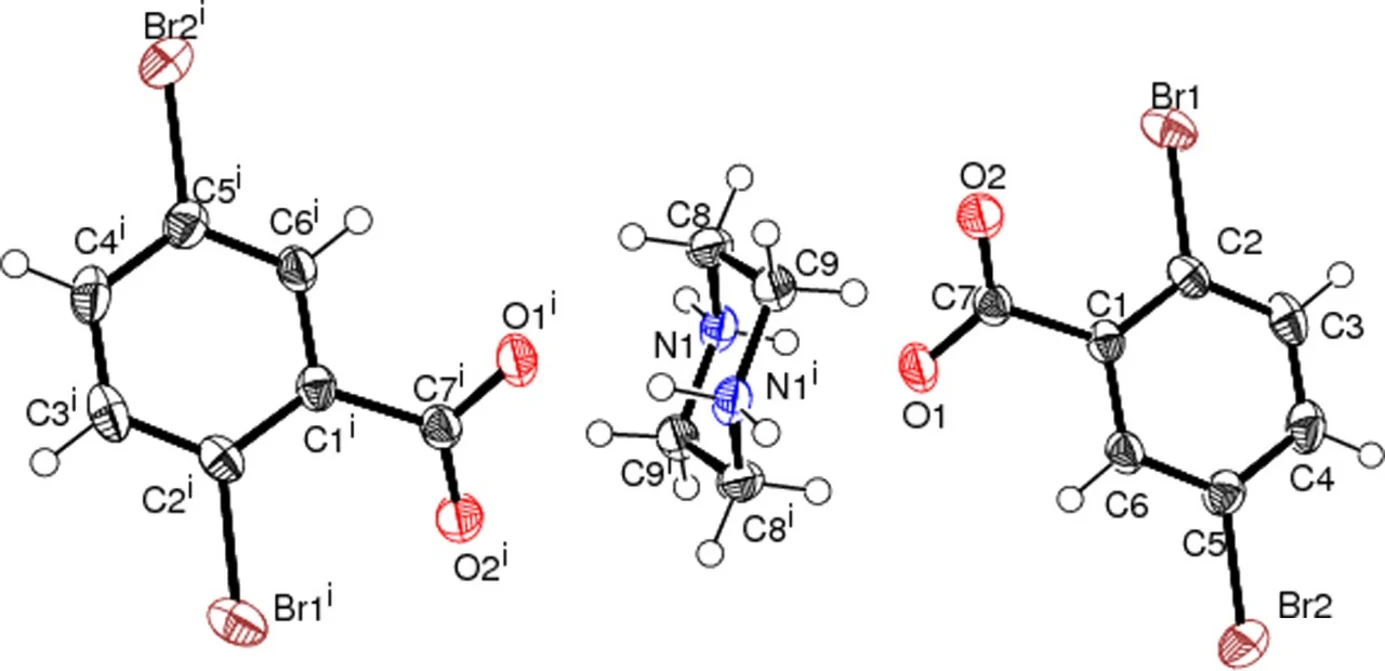
The mol­ecular structure of the title mol­ecular salt (I) showing the atom-labeling scheme. Displacement ellipsoids are drawn at the 50% probability level. Symmetry code: (i) −*x* + 2, −*y* + 1, −*z* + 1).

The title salt (I) crystallizes in the monoclinic space group *P*2_1_/*c*. The asymmetric unit comprises one-half of a piperazine-1,4-diium dication, located about an inversion center, and one 2,5-di­bromo­benzoate anion (Fig. 1 ▸). In the dication, both nitro­gen atoms (N1 and its inversion-related equivalent) of the piperazine ring are protonated, while the 2,5-di­bromo­benzoic acid is deprotonated at the carboxyl group. Proton transfer from acid to piperazine mol­ecule leads to the formation of the ionic salt. Charge neutrality is achieved by one dication for every two monobasic 2,5-di­bromo­benzoate anions in the crystal structure.

The piperazine ring adopts a chair conformation, with puckering parameters *Q* = 0.575 (3) Å, θ = 0°, and φ = 0°. The geometric parameters, including bond lengths, bond angles, and torsion angles, for both dication and 2,5-di­bromo­benzoate anion are comparable to those reported for related structures.

In the crystal, the piperazine-1,4-diium cation and two 2,5-di­bromo­benzoate anions are connected via N1—H1*A*⋯O1 hydrogen bonding, complemented by additional C—H⋯Br inter­actions (Table 1 ▸), contributing to the cohesion of the crystal structure.

## 3. Supra­molecular features

Both O atoms of the carboxyl­ate group and Br atoms act as hydrogen-bond acceptors in a series of N—H⋯O, N—H⋯Br, and C—H⋯Br inter­actions within the crystal structure (Table 1 ▸). The carboxyl­ate O atoms (O1 and O2) form hydrogen bonds with the protonated N atoms of the piperazine-1,4-diium dication via N1—H1*A*⋯O1 and N1—H1*B*⋯O2^v^ [2.655 (3) Å and 2.755 (3) Å, respectively; symmetry code: (v) −*x* + 2, *y* − 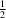, −*z* + 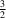]).

**Figure 2:**
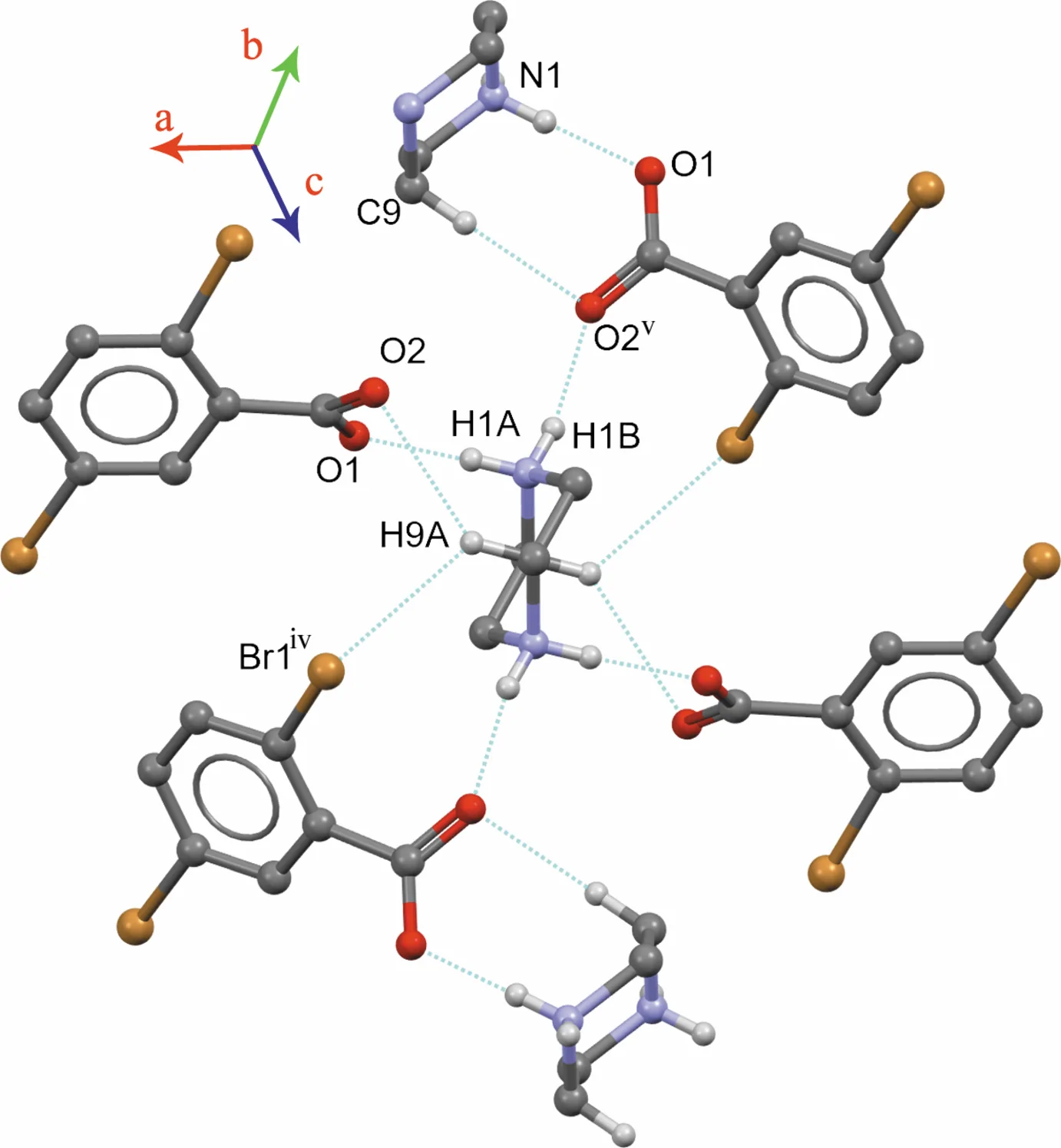
Part of the crystal structure of (I), showing the piperazine-1,4-diium cation linked to four neighboring anions *via* N1—H1*A*⋯O1, N1—H1*B*⋯O2, C9—H9*A*⋯O2, and C9—H9*A*⋯Br1 hydrogen bonds, forming a mol­ecular double layer parallel to the *bc* plane. Symmetry codes as in Table 1.

Each piperazine-1,4-diium cation is linked to four neighbouring anions through N—H⋯O, C—H⋯O/Br inter­actions [N1—H1*A*⋯O1, N1—H1*B*⋯O2^v^, C9—H9*A*⋯O2 and C9—H9*A*⋯Br1^iv^; symmetry code: (iv) *x*, −*y* + 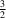, *z* − 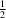], generating a mol­ecular double layer parallel to the *bc* plane (Fig. 2 ▸).

**Figure 3:**
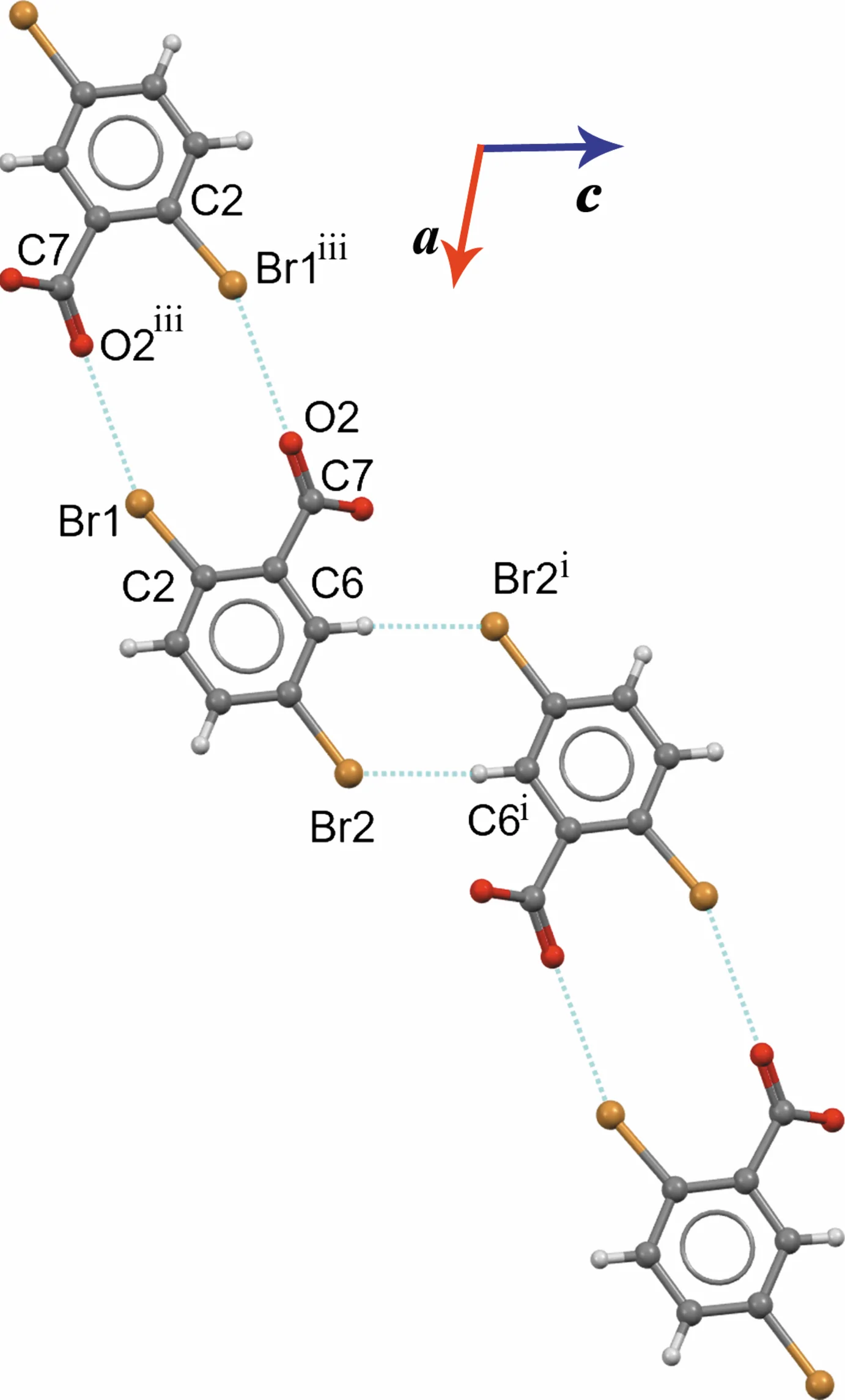
Part of the crystal structure of (I), showing a mol­ecular chain formed *via* C6—H6⋯Br2 and C=O⋯Br inter­actions, viewed down the *b* axis. Symmetry codes as in Table 1.

Two neighbouring anions are further connected via a C6—H6⋯Br2^i^ hydrogen bond [symmetry code: (i) −*x* + 1, −*y* + 1, −*z* + 1], forming a centrosymmetric 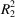
(8) ring motif. In addition, adjacent motifs are linked through short C2=O2⋯Br1(−*x* + 2, −*y* + 1, −*z* + 2) contacts [3.3183 (2) Å], resulting in the formation of a mol­ecular chain lying in the *ac* plane (Fig. 3 ▸).

**Figure 4:**
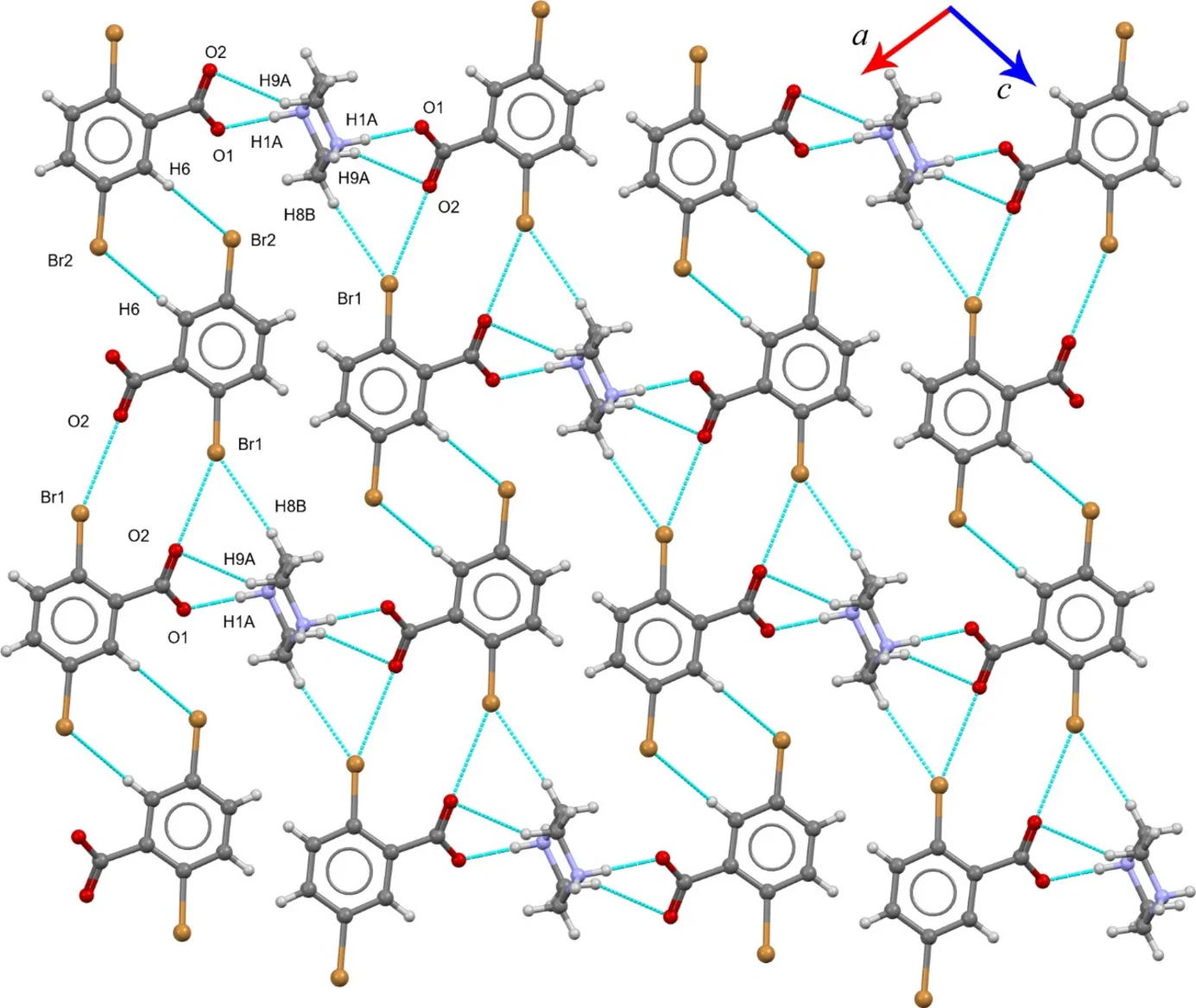
Part of the crystal structure of (I), showing a two-dimensional mol­ecular layer formed *via* N1—H1*A*⋯O1, C9—H9*A*⋯O2, C6—H6⋯Br2, C8—H8*B*⋯Br1, and C=O⋯Br1 inter­actions, viewed down the *b* axis.

The two-dimensional mol­ecular layers are constructed through the combined N—H⋯O, C—H⋯O/Br inter­actions [N1—H1*A*⋯O1, C9—H9*A*⋯O2, C6—H6⋯Br, and C8—H8B⋯Br1] together with C2=O2⋯Br1 contacts, extending parallel to the *ac* plane (Fig. 4 ▸). These layers are further inter­connected by additional N—H⋯O/Br, and C—H⋯O/Br inter­actions [C8—H8*A*⋯Br2^ii^, C9—H9*A*⋯Br1^iv^, C9—H9*B*⋯Br2^ii^, N1—H1*B*⋯O2^i^; N1—H1*B*⋯Br1^v^], as well as a C—Br⋯π inter­action involving the phenyl ring centroid [C5—Br2⋯*Cg*1(1 − *x*, −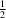 + *y*, 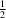 − *z*) = 3.8107 (13) Å], leading to the formation of a three-dimensional supra­molecular framework.

## 4. Hirshfeld surface analysis

Hirshfeld surfaces (HS) and two-dimensional fingerprint plots (2D-FP) were generated using *Crystal Explorer 17.5* (Turner *et al.*, 2017 ▸) for both the cation and the anion of the title salt. These analyses enable visualization and qu­anti­fication of inter­molecular inter­actions within the crystal packing (McKinnon *et al.*, 1998 ▸, 2004 ▸, 2007 ▸; Spackman & Jayatilaka, 2009 ▸; Spackman & McKinnon, 2002 ▸). The HS mapped over *d*_e_ (based on the distance from the surface to the nearest external atom) and *d*_i_ (distance from the nearest inter­nal atom to the surface), facilitates identification of close contacts and their relative contributions. Variations in colors and intensities on the HS indicate short (red) and long (blue) contacts, highlight the significance of specific inter­molecular inter­actions (Venkatesan *et al.*, 2015 ▸, 2016*a* ▸,*b* ▸). The decomposed 2D-FP plots provide a qu­anti­tative breakdown of individual contact contributions.

**Figure 5:**
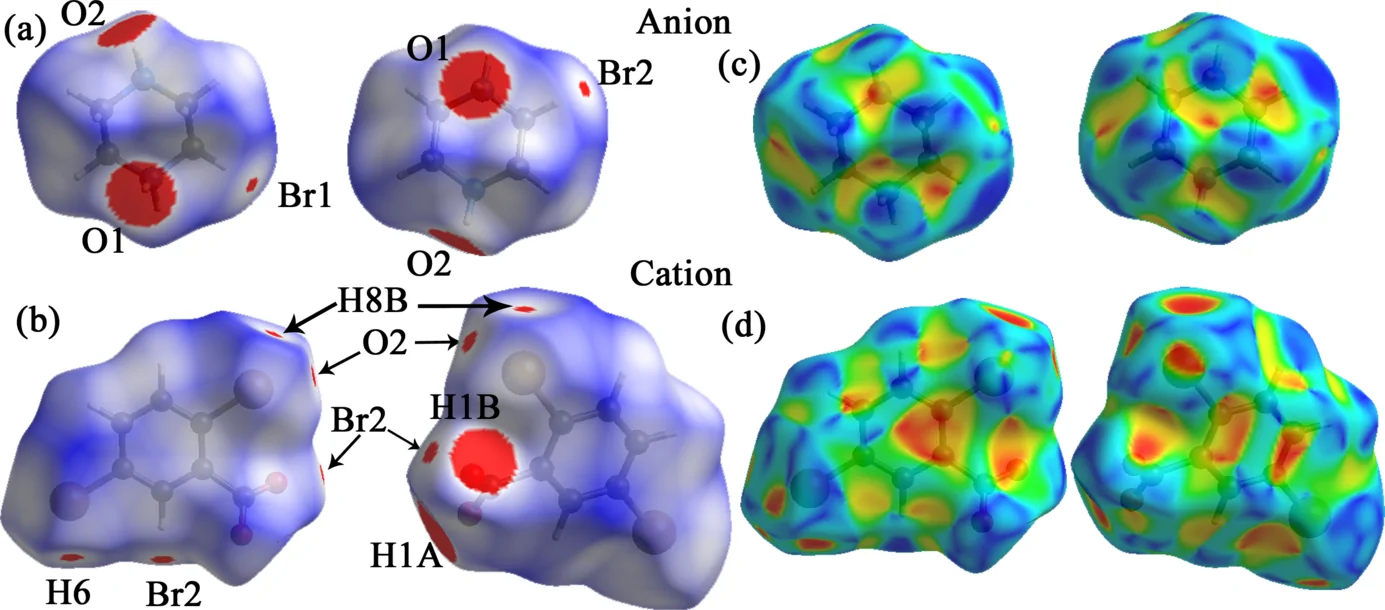
Hirshfeld surface of the cation and anion in the title salt shown in two orientations, mapped with (*a*) and (*b*) *d_norm_* and (*c*) and (*d*) shape-index (SI).

The HS mapped over *d*_norm_ in the range −0.75 to 1.1425 a.u. is shown (front and back) for both the dication and anion in Fig. 5 ▸*a*,*b*. Prominent bright-red spots correspond to strong hydrogen-bonding inter­actions, notably N1—H1*A*⋯O1 and N1—H1*B*⋯O2. Weaker red regions are associated with C—H⋯Br inter­actions, such as C6—H6⋯Br2 and C8—H8*B*⋯Br1. The C7=O2⋯Br1 contact is also visible as a weak red spot, suggesting a weak non-covalent inter­action. The shape-index (SI) surfaces (Fig. *5c*,*d*) do not display complementary red and blue triangular patterns over the aromatic rings, confirming the absence of π–π stacking inter­actions in the crystal structure.

**Figure 6:**
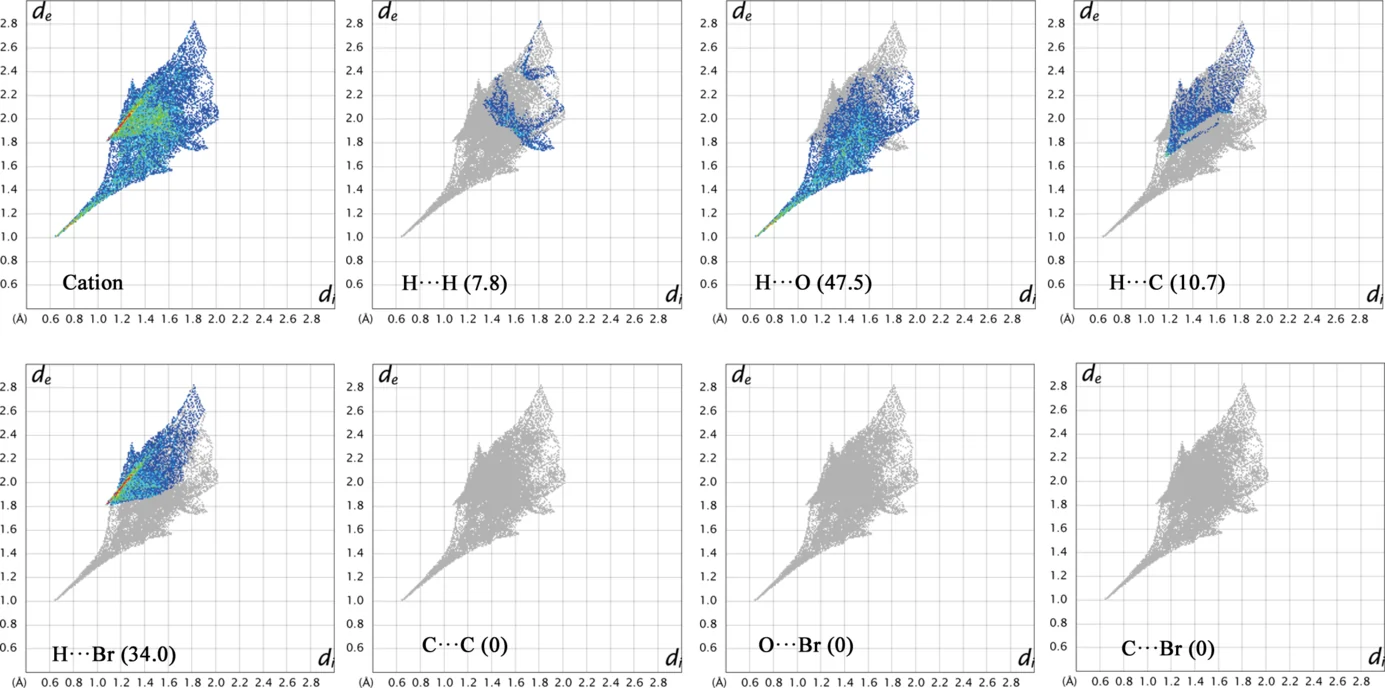
The full and decomposed two-dimensional fingerprint plots for the cation in the title salt (I), showing the relative contributions of the respective reciprocal contacts.

The full and decomposed 2D-FP plots for the dication are shown in Fig. 6 ▸ ▸, with the corresponding percentage contributions summarized in Fig. 8 ▸. The dominant inter­molecular contacts for the dication are H⋯O/O⋯H inter­actions, contributing 47.5% and appearing as a sharp spike at *d*_e_ + *d_i_* = 1.6 Å. The next major contribution arises from H⋯Br/Br⋯H contacts (34.0%), represented by a weak spike around *d*_e_ + *d*_i_ = 3.0 Å. Minor contributions include H⋯C/C⋯H (10.7%) and H⋯H (7.8%) contacts.

**Figure 7:**
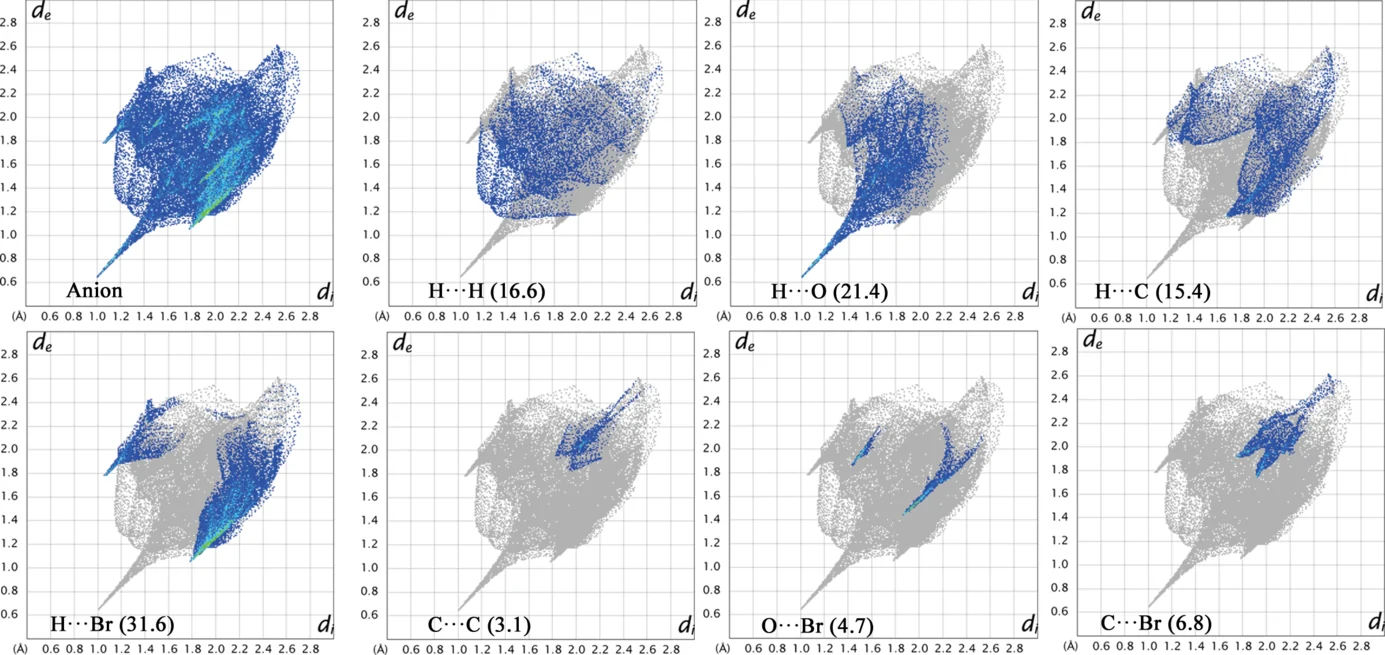
The full and decomposed two-dimensional fingerprint plots for the anion in the title salt (I), showing the relative contributions of the respective reciprocal contacts.

**Figure 8:**
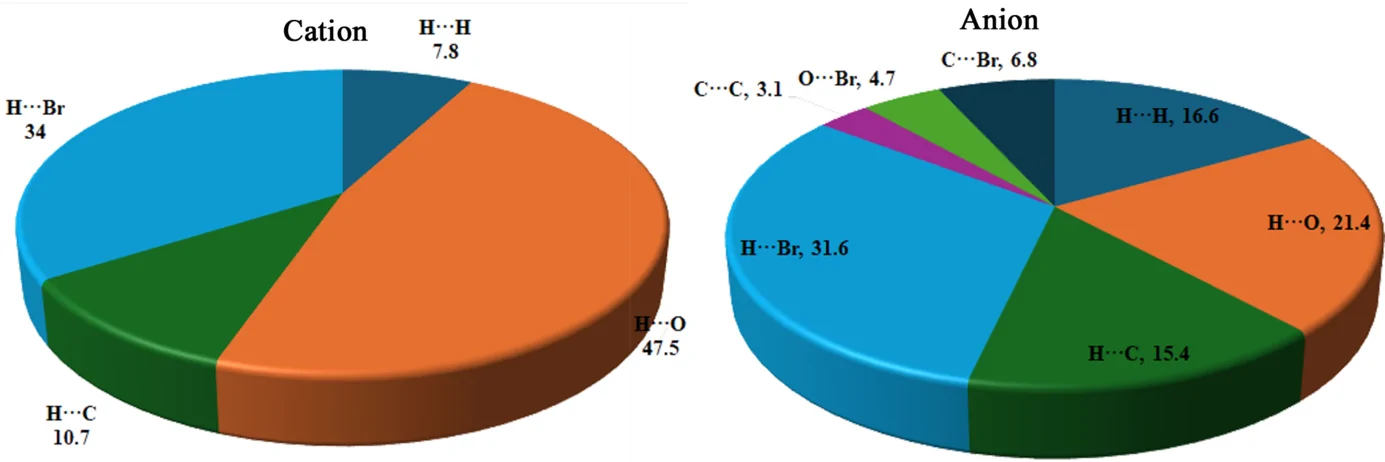
Pie charts showing the relative contributions of various inter­molecular contacts to the Hirshfeld surfaces of the cation and anion in the title salt (I).

In contrast, the anion (Fig. 7 ▸) is dominated by H⋯Br/Br⋯H inter­actions (31.6%), which appear as an asymmetric wing-like pattern at *d*_e_ + *d*_i_ = 2.9 Å. The contribution of H⋯O/O⋯H contacts is 21.4%, and these still appear as sharp spikes near *d*_e_ + *d*_i_ = 1.6 Å. This reduction is compensated by significant contributions from H⋯H (16.6%), H⋯C/C⋯H (15.4%), Br⋯O/O⋯Br (4.7%), and C⋯Br/Br⋯C (6.8%) inter­actions. These contacts arise from the involvement of Br atom in inter­actions such as C7=O2⋯Br1 and C5—Br2⋯*Cg*1, which are absent in the cation (where *Cg*1 is the centroid of the C1–C6 ring). The C⋯C contacts contribute only 3.1% in the anion and are negligible in the cation, confirming the absence of π–π inter­actions, in agreement with the shape-index analysis. Furthermore, the lack of significant features beyond *d*_e_ + *d*_i_ = 2.4 Å suggests that H⋯H inter­actions play only a minor role in consolidating the crystal structure. The contributions of various inter­actions in the cation and anion are compared in Fig. 8 ▸.

## 5. Database survey

A search of the Cambridge Structural Database (CSD, Version 5.45, update of June 2024; Groom *et al.*, 2016 ▸) using Conquest (Bruno *et al.*, 2002 ▸) for piperazine-1,4-diium salts with substituted benzoic acids, including chloro-, amino-, nitro-, hy­droxy-, and polycarb­oxy benzoic acids was performed. In particular, the crystal structures of piperazinediium bis­(2/3/4-chloro­benzoate) (CSD refcodes ALOQIC, ALOQOI, ALOQUO; Chen & Peng 2011 ▸), piperazinediium bis­(2-amino­benzoate) (ALORAV; Chen & Peng 2011 ▸); piperazinediium bis­(4-amino­benzoate) (HEZTAI and HEZTAI01, Moulton *et al.*, 2007 ▸; Chen & Peng 2011 ▸) have been reported, and like the title compound, contain the piperazinediium ion.

## 6. Synthesis and crystallization

A 250 mL round-bottom flask was charged with piperazine (1 g, 11.6 mmol) and 2,5-di­bromo­benzoic acid (6.5 g, 23.2 mmol) in a 1:2 molar ratio. The mixture was dissolved in 50 mL of methanol at room temperature and stirred continuously for 6 h. The resulting homogeneous solution was filtered using Whatman filter paper and left undisturbed in a dust-free environment to evaporate slowly at room temperature. After 10 days, red block-like crystals, with m.p. 458 K, suitable for single-crystal X-ray diffraction were obtained.
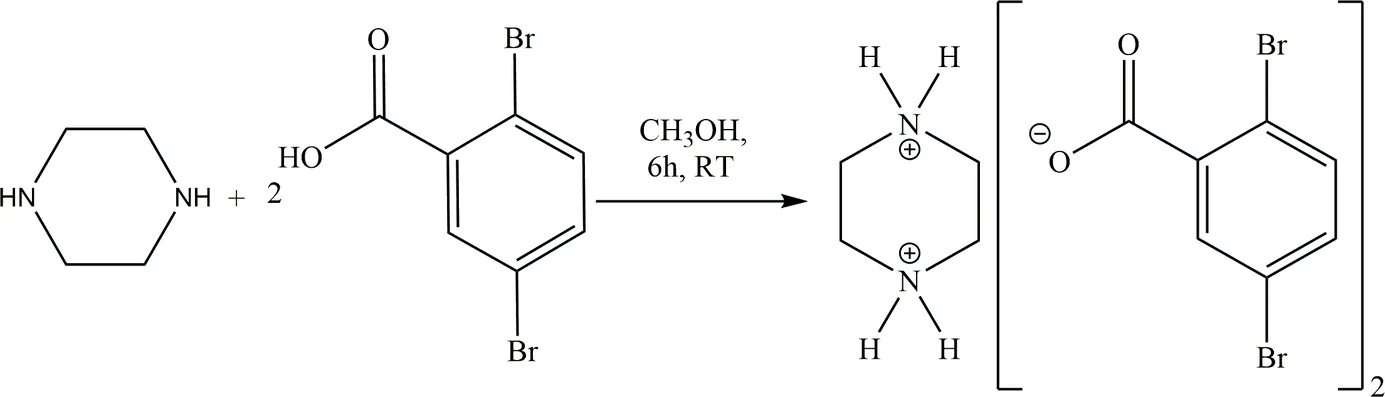


## 7. Refinement

Crystal data, data collection and structure refinement details are summarized in Table 2 ▸. The N-bound H atoms were located in a difference-Fourier map and refined with restrained N—H and H⋯H distances. C-bound H atoms were positioned geometrically (0.93–0.97 Å) and refined as riding with *U*_iso_(H) = 1.2*U*_eq_(C).

## Supplementary Material

Crystal structure: contains datablock(s) I. DOI: 10.1107/S205698902600798X/ej2013sup1.cif

Structure factors: contains datablock(s) I. DOI: 10.1107/S205698902600798X/ej2013Isup3.hkl

Supporting information file. DOI: 10.1107/S205698902600798X/ej2013Isup3.cml

Supporting information file. DOI: 10.1107/S205698902600798X/ej2013Isup3.cml

CCDC reference: 2578701

Additional supporting information:  crystallographic information; 3D view; checkCIF report

## Figures and Tables

**Table 1:** Hydrogen-bond geometry (Å, °)

*D*—H⋯*A*	*D*—H	H⋯*A*	*D*⋯*A*	*D*—H⋯*A*
N1—H1*A*⋯O1	0.87 (2)	1.79 (2)	2.655 (3)	178 (4)
C9—H9*A*⋯O2	0.97	2.67	3.440 (4)	136
C6—H6⋯Br2^i^	0.93	2.98	3.855 (3)	157
C8—H8*A*⋯Br2^ii^	0.97	3.07	3.661 (3)	121
C8—H8*B*⋯Br1^iii^	0.97	3.00	3.967 (3)	173
C9—H9*A*⋯Br1^iv^	0.97	3.00	3.655 (3)	126
C9—H9*B*⋯Br2^ii^	0.97	3.10	3.781 (3)	128
N1—H1*B*⋯O2^v^	0.86 (2)	1.93 (2)	2.755 (3)	160 (3)
N1—H1*B*⋯Br1^v^	0.86 (2)	3.06 (3)	3.598 (2)	123 (3)

Symmetry codes: (i) 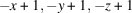; (ii) 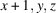; (iii) 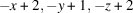; (iv) 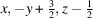; (v) 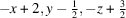.

**Table 2:** Experimental details

Crystal data	
Chemical formula	0.5C_4_H_12_N_2_^2+^·C_7_H_3_Br_2_O_2_^−^
*M*_r_	322.99
Crystal system, space group	Monoclinic, *P*2_1_/*c*
Temperature (K)	298
*a*, *b*, *c* (Å)	11.7467 (5), 7.9755 (3), 11.3340 (4)
β (°)	101.052 (1)
*V* (Å^3^)	1042.14 (7)
*Z*	4
Radiation type	Mo *K*α
μ (mm^−1^)	7.75
Crystal size (mm)	0.23 × 0.20 × 0.15

Data collection	
Diffractometer	Bruker D8 VENTURE diffractometer equipped with PHOTON II detector
Absorption correction	Multi-scan (*SADABS*; Krause *et al.*, 2015 ▸)
*T*_min_, *T*_max_	0.462, 0.746
No. of measured, independent and observed [*I* > 2σ(*I*)] reflections	28846, 2565, 2133
*R*_int_	0.056
(sin θ/λ)_max_ (Å^−1^)	0.667

Refinement	
*R*[*F*^2^ > 2σ(*F*^2^)], *wR*(*F*^2^), *S*	0.033, 0.073, 1.07
No. of reflections	2565
No. of parameters	135
No. of restraints	3
H-atom treatment	H atoms treated by a mixture of independent and constrained refinement
Δρ_max_, Δρ_min_ (e Å^−3^)	0.93, −0.75

Computer programs: *APEX4*, *SAINT* and *XPREP* (Bruker, 2021 ▸), *SHELXT* (Sheldrick, 2015*a* ▸), *SHELXL2018/3* (Sheldrick, 2015*b* ▸), *ORTEP-3 for Windows* (Farrugia, 2012 ▸), *PLATON* (Spek, 2020 ▸) and *Mercury* (Macrae *et al.*, 2020 ▸).

## supplementary crystallographic information

### Piperazine-1,4-diium bis (2,5-dibromobenzoate) Crystal data

**Table:**

0.5C_4_H_12_N_2_^2+^·C_7_H_3_Br_2_O_2_^−^	*F*(000) = 624
*M**_r_* = 322.99	*D*_x_ = 2.059 Mg m^−^^3^
Monoclinic, *P*2_1_/*c*	Mo *K*α radiation, λ = 0.71073 Å
*a* = 11.7467 (5) Å	Cell parameters from 9898 reflections
*b* = 7.9755 (3) Å	θ = 3.1–27.9°
*c* = 11.3340 (4) Å	µ = 7.75 mm^−^^1^
β = 101.052 (1)°	*T* = 298 K
*V* = 1042.14 (7) Å^3^	Block, colourless
*Z* = 4	0.23 × 0.20 × 0.15 mm

### Piperazine-1,4-diium bis (2,5-dibromobenzoate) Data collection

**Table:**

Bruker D8 VENTURE diffractometer equipped with PHOTON II detector	2133 reflections with *I* > 2σ(*I*)
Radiation source: fine-focus sealed tube	*R*_int_ = 0.056
ω and phi scans	θ_max_ = 28.3°, θ_min_ = 3.4°
Absorption correction: multi-scan (SADABS; Krause *et al.*, 2015)	*h* = −15→15
*T*_min_ = 0.462, *T*_max_ = 0.746	*k* = −10→10
28846 measured reflections	*l* = −15→15
2565 independent reflections	

### Piperazine-1,4-diium bis (2,5-dibromobenzoate) Refinement

**Table:**

Refinement on *F*^2^	3 restraints
Least-squares matrix: full	Hydrogen site location: mixed
*R*[*F*^2^ > 2σ(*F*^2^)] = 0.033	H atoms treated by a mixture of independent and constrained refinement
*wR*(*F*^2^) = 0.073	*w* = 1/[σ^2^(*F*_o_^2^) + (0.0208*P*)^2^ + 1.9785*P*] where *P* = (*F*_o_^2^ + 2*F*_c_^2^)/3
*S* = 1.07	(Δ/σ)_max_ = 0.001
2565 reflections	Δρ_max_ = 0.93 e Å^−^^3^
135 parameters	Δρ_min_ = −0.75 e Å^−^^3^

### Piperazine-1,4-diium bis (2,5-dibromobenzoate) Special details

**Table:**

Geometry. All esds (except the esd in the dihedral angle between two l.s. planes) are estimated using the full covariance matrix. The cell esds are taken into account individually in the estimation of esds in distances, angles and torsion angles; correlations between esds in cell parameters are only used when they are defined by crystal symmetry. An approximate (isotropic) treatment of cell esds is used for estimating esds involving l.s. planes.

### Piperazine-1,4-diium bis (2,5-dibromobenzoate) Fractional atomic coordinates and isotropic or equivalent isotropic displacement parameters (Å^2^)

**Table:**

	*x*	*y*	*z*	*U*_iso_*/*U*_eq_	
C1	0.7131 (2)	0.5605 (3)	0.7922 (2)	0.0246 (5)	
C2	0.6993 (3)	0.5879 (4)	0.9100 (2)	0.0275 (6)	
C3	0.5922 (3)	0.6344 (4)	0.9357 (3)	0.0359 (7)	
H3	0.584948	0.651873	1.015030	0.043*	
C4	0.4973 (3)	0.6545 (4)	0.8449 (3)	0.0365 (7)	
H4	0.426181	0.687138	0.861885	0.044*	
C5	0.5096 (2)	0.6255 (4)	0.7280 (3)	0.0306 (6)	
C6	0.6150 (2)	0.5787 (4)	0.7012 (3)	0.0275 (6)	
H6	0.620851	0.559147	0.621684	0.033*	
C7	0.8252 (2)	0.5091 (4)	0.7527 (2)	0.0263 (6)	
C8	1.0922 (3)	0.4660 (4)	0.6006 (3)	0.0312 (6)	
H8A	1.155531	0.435851	0.561002	0.037*	
H8B	1.120077	0.459490	0.686723	0.037*	
C9	1.0533 (3)	0.6428 (4)	0.5665 (3)	0.0308 (6)	
H9A	0.994383	0.676443	0.611181	0.037*	
H9B	1.118506	0.718859	0.587074	0.037*	
N1	0.9947 (2)	0.3469 (3)	0.5648 (2)	0.0278 (5)	
O1	0.81248 (19)	0.4115 (3)	0.6644 (2)	0.0428 (6)	
O2	0.91953 (18)	0.5667 (3)	0.8059 (2)	0.0386 (5)	
Br1	0.82116 (3)	0.54666 (5)	1.04365 (3)	0.04140 (11)	
Br2	0.37973 (3)	0.64277 (5)	0.60002 (3)	0.04320 (11)	
H1A	0.936 (2)	0.367 (4)	0.599 (3)	0.049 (11)*	
H1B	1.021 (3)	0.249 (3)	0.588 (3)	0.044 (10)*	

### Piperazine-1,4-diium bis (2,5-dibromobenzoate) Atomic displacement parameters (Å^2^)

**Table:**

	*U*^11^	*U*^22^	*U*^33^	*U*^12^	*U*^13^	*U*^23^
C1	0.0252 (13)	0.0234 (13)	0.0270 (13)	−0.0029 (10)	0.0095 (10)	−0.0019 (11)
C2	0.0349 (15)	0.0261 (14)	0.0226 (12)	−0.0049 (11)	0.0081 (11)	−0.0014 (11)
C3	0.0446 (18)	0.0386 (18)	0.0296 (15)	−0.0032 (14)	0.0198 (13)	−0.0064 (13)
C4	0.0317 (15)	0.0417 (18)	0.0409 (17)	0.0007 (13)	0.0193 (13)	−0.0041 (14)
C5	0.0256 (14)	0.0322 (16)	0.0350 (15)	−0.0008 (12)	0.0082 (12)	−0.0010 (13)
C6	0.0286 (14)	0.0318 (15)	0.0240 (13)	−0.0006 (11)	0.0097 (11)	−0.0026 (11)
C7	0.0280 (14)	0.0286 (14)	0.0236 (12)	0.0003 (11)	0.0081 (11)	0.0003 (11)
C8	0.0263 (14)	0.0376 (17)	0.0284 (14)	0.0014 (12)	0.0018 (11)	0.0021 (13)
C9	0.0307 (14)	0.0325 (15)	0.0298 (14)	0.0005 (12)	0.0075 (11)	−0.0036 (12)
N1	0.0275 (12)	0.0295 (13)	0.0290 (12)	0.0058 (10)	0.0117 (10)	0.0042 (10)
O1	0.0286 (11)	0.0612 (15)	0.0404 (12)	−0.0026 (10)	0.0111 (9)	−0.0251 (12)
O2	0.0274 (11)	0.0489 (14)	0.0395 (12)	−0.0045 (10)	0.0063 (9)	−0.0148 (11)
Br1	0.0485 (2)	0.0501 (2)	0.02394 (15)	−0.00516 (15)	0.00278 (12)	0.00032 (14)
Br2	0.02605 (16)	0.0556 (2)	0.0469 (2)	0.00428 (14)	0.00450 (13)	−0.00056 (16)

### Piperazine-1,4-diium bis (2,5-dibromobenzoate) Geometric parameters (Å, º)

**Table:**

C1—C2	1.392 (4)	C7—O2	1.243 (4)
C1—C6	1.399 (4)	C7—O1	1.254 (4)
C1—C7	1.526 (4)	C8—N1	1.484 (4)
C2—C3	1.395 (4)	C8—C9	1.510 (4)
C2—Br1	1.903 (3)	C8—H8A	0.9700
C3—C4	1.374 (5)	C8—H8B	0.9700
C3—H3	0.9300	C9—N1^i^	1.488 (4)
C4—C5	1.379 (4)	C9—H9A	0.9700
C4—H4	0.9300	C9—H9B	0.9700
C5—C6	1.382 (4)	N1—H1A	0.870 (18)
C5—Br2	1.897 (3)	N1—H1B	0.864 (17)
C6—H6	0.9300		
			
C2—C1—C6	117.4 (3)	O1—C7—C1	115.0 (2)
C2—C1—C7	126.1 (3)	N1—C8—C9	110.4 (2)
C6—C1—C7	116.5 (2)	N1—C8—H8A	109.6
C1—C2—C3	121.2 (3)	C9—C8—H8A	109.6
C1—C2—Br1	121.9 (2)	N1—C8—H8B	109.6
C3—C2—Br1	116.8 (2)	C9—C8—H8B	109.6
C4—C3—C2	120.6 (3)	H8A—C8—H8B	108.1
C4—C3—H3	119.7	N1^i^—C9—C8	110.2 (2)
C2—C3—H3	119.7	N1^i^—C9—H9A	109.6
C3—C4—C5	118.7 (3)	C8—C9—H9A	109.6
C3—C4—H4	120.6	N1^i^—C9—H9B	109.6
C5—C4—H4	120.6	C8—C9—H9B	109.6
C4—C5—C6	121.3 (3)	H9A—C9—H9B	108.1
C4—C5—Br2	120.3 (2)	C8—N1—C9^i^	111.2 (2)
C6—C5—Br2	118.3 (2)	C8—N1—H1A	113 (3)
C5—C6—C1	120.8 (3)	C9^i^—N1—H1A	105 (3)
C5—C6—H6	119.6	C8—N1—H1B	107 (3)
C1—C6—H6	119.6	C9^i^—N1—H1B	113 (3)
O2—C7—O1	125.1 (3)	H1A—N1—H1B	107 (2)
O2—C7—C1	119.8 (3)		
			
C6—C1—C2—C3	−1.0 (4)	Br2—C5—C6—C1	−178.6 (2)
C7—C1—C2—C3	−179.9 (3)	C2—C1—C6—C5	1.3 (4)
C6—C1—C2—Br1	173.8 (2)	C7—C1—C6—C5	−179.8 (3)
C7—C1—C2—Br1	−5.0 (4)	C2—C1—C7—O2	−37.1 (4)
C1—C2—C3—C4	−0.1 (5)	C6—C1—C7—O2	144.1 (3)
Br1—C2—C3—C4	−175.2 (3)	C2—C1—C7—O1	144.3 (3)
C2—C3—C4—C5	1.0 (5)	C6—C1—C7—O1	−34.5 (4)
C3—C4—C5—C6	−0.7 (5)	N1—C8—C9—N1^i^	−56.7 (3)
C3—C4—C5—Br2	177.4 (2)	C9—C8—N1—C9^i^	57.4 (3)
C4—C5—C6—C1	−0.4 (5)		

Symmetry code: (i) −*x*+2, −*y*+1, −*z*+1.

### Piperazine-1,4-diium bis (2,5-dibromobenzoate) Hydrogen-bond geometry (Å, º)

**Table:**

*D*—H···*A*	*D*—H	H···*A*	*D*···*A*	*D*—H···*A*
N1—H1*A*···O1	0.87 (2)	1.79 (2)	2.655 (3)	178 (4)
C9—H9*A*···O2	0.97	2.67	3.440 (4)	136
C6—H6···Br2^ii^	0.93	2.98	3.855 (3)	157
C8—H8*A*···Br2^iii^	0.97	3.07	3.661 (3)	121
C8—H8*B*···Br1^iv^	0.97	3.00	3.967 (3)	173
C9—H9*A*···Br1^v^	0.97	3.00	3.655 (3)	126
C9—H9*B*···Br2^iii^	0.97	3.10	3.781 (3)	128
N1—H1*B*···O2^vi^	0.86 (2)	1.93 (2)	2.755 (3)	160 (3)
N1—H1*B*···Br1^vi^	0.86 (2)	3.06 (3)	3.598 (2)	123 (3)

Symmetry codes: (ii) −*x*+1, −*y*+1, −*z*+1; (iii) *x*+1, *y*, *z*; (iv) −*x*+2, −*y*+1, −*z*+2; (v) *x*, −*y*+3/2, *z*−1/2; (vi) −*x*+2, *y*−1/2, −*z*+3/2.
